# Computational Ecology: From the Complex to the Simple and Back

**DOI:** 10.1371/journal.pcbi.0010018

**Published:** 2005-07-29

**Authors:** Mercedes Pascual

In 1958, when ecology was a young science and mathematical models for ecological systems were in their infancy, Elton [1] wrote of the “neolithic days of animal ecology, that is to say about twenty-five years ago.” Acknowledging the influence of Lotka and Volterra, he noted, “Being mathematicians, they did not attempt to contemplate a whole food-chain with all the complications of five stages. They took two: a predator and its prey.”

Today, in the era of computational ecological modeling, deterministic systems for two variables—and even a whole food chain—appear like simple idealizations well removed from the complexity of nature. We now consider predator–prey interactions as “consumer–resource” interactions embedded within the large ecological networks that underlie biodiversity (Figure 1) [2]. Consequently, the scale of the problems we model has grown to reflect the world as we now need to observe it. For example, the interplay between ecosystem dynamics and the physical environment that influences global change occurs over a tremendous range of spatial and organizational scales (e.g., [3]). Similarly, the population dynamics of the transmission of infectious diseases often involve spatial or social networks with large numbers of individuals, but the interactions of each individual involve only a subset of the network and can span from local to global distances (e.g., [4–6]).

**Figure 1:**
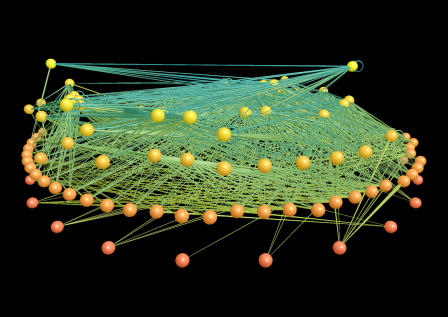
The Network of Trophic Interactions for Little Rock Lake, Wisconsin Figure shows 997 feedings links (lines) among 92 taxa (nodes) [2]. The node color indicates the trophic level of the taxon, including (from bottom to top) algae, zooplankton, insects, and fishes; the link color corresponds to the type of feeding link, including herbivory and primary and secondary carnivory. This image was produced using FoodWeb3D software written by R. J. Williams and provided by the Pacific Ecoinformatics and Computational Ecology Lab (www.foodwebs.org).

These examples illustrate the current view of ecological systems as complex adaptive systems [7,8]. Complex adaptive systems are distinguished not only by the multiplicity of components within them, but also by interactions that can be local or distributed among these components and whose rates vary as nonlinear functions of the state of the system itself. One obvious role of computation in the science of complex systems is simply one of synthesis: to reconstruct the whole from the parts as we learn more and more about the components and their interactions. There are obvious limitations to this approach, evident in the famous image of those imperial cartographers who produced a map of the empire of the same size as the empire itself [9]. I argue here that an alternative and more useful role of computation is to address questions on the relationship between dynamics at different temporal, spatial, and organizational scales, that is, to address the importance of variability at small, local scales to the dynamics of aggregated quantities measured at large, global scales. If small-scale “details” matter, we need to ask how much complexity we need to incorporate into large-scale models if we seek to both understand and predict the dynamics of global quantities.

Is it possible to incorporate the effect of small-scale variability without resorting to the “brute force” approach of using higher and higher resolution? I start with examples from theoretical ecology that illustrate problems and approaches related to these scaling questions; I then present more specific examples related to global change and ecosystem dynamics, and end with a series of related problems on the dynamics of large food webs, the ultimate networks of ecological interactions.

## From Individuals to Populations: How Local Effects Translate into Global Results

Lotka–Volterra equations and their many descendants assume that individuals are well mixed and interact at mean population abundances. They are mean-field equations that use the mass-action law to describe the dynamics of interacting populations, and ignore both the scale of individual interactions and their spatial distribution. A key question therefore is: can the spatial variability generated at small, individual scales influence the dynamics at larger, population scales? If so, can the effect of smaller scales be represented by simply modifying mean-field models?

**Figure 2:**
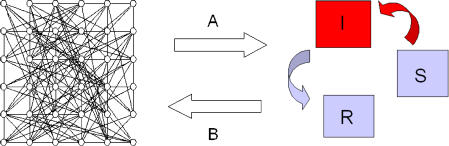
Bridging Dynamics across Organizational Scales On the left is a detailed model in which individual interactions in a network are described explicitly. On the right, typical “mean field” models aggregate the population into compartments (here for the three subpopulations of susceptible, infected, and recovered individuals in the dynamics of an infectious disease with permanent immunity). Computational approaches can help us understand the relationship between dynamics at these two different scales, from the individual to the population level. We can start with a stochastic individual-based model and develop approximations that simplify it (A). From this process, we can learn about the opposite direction of formulating simple models directly without sufficient knowledge to first specify the detailed interactions and components (B). These simple models represent implicitly the effect of smaller scale variability.

Stochastic models such as interacting particle systems [10] can help us examine approaches for scaling up individual-based dynamics [4,11–14]. One of the most useful lessons learned from scaling up detailed models is also pertinent to the related but opposite problem: the formulation of simple models for global variables that still account for the effects of local interactions but do not represent them explicitly (Figure 2). In particular, can we formulate these systems without having to first simplify detailed models? The problem with simplification is that it assumes we know about the components and interactions at “microscopic” levels, but, unfortunately, this information is not often available. For example, we may know that the social network underlying the propagation of an infectious disease is important, but we may not know all the interactions and the specific contacts that led to transmission of the disease from one individual to another. If we were to start with an aggregated model at the population level (one in which the population is aggregated into a few variables describing the total number of infected, susceptible, and immune individuals), how would we formulate it to incorporate variability at spatial or organizational levels that are not explicitly represented?

Recent findings on individual-based models for predator–prey dynamics in a spatial lattice indicate that simpler, low-dimensional models can still be applied at the population level [13,14]. Specifically, the temporal dynamics of global population abundances, aggregated over the whole lattice, can be approximated by mean-field-type equations in which the functional forms specifying the rates of growth and interaction have been modified as power functions. Similar results hold for disease dynamics on spatial and social networks ([15]; M. M. Maule and J. A. N. Filipe, unpublished data; M. Roy and M. Pascual, unpublished data). The rates of transmission of the original mean-field equation are modified to account for deviations from mass action by incorporating nonlinear mixing terms between susceptible and infected populations in which global abundances are raised to a power. Thus, the effect of interactions at local, individual scales can be represented implicitly by changing the shape of the functions describing interactions at global, population levels; that is, the modified framework is structured as if mass action applied when in fact it does not, yet the subtleties of nonrandom mixing are captured at the higher scale. The generality of these findings and the reasons why power-law functional forms yield successful approximations remain to be determined. Another approach based on moment closure techniques has been applied to simplify detailed models by incorporating the effects of variances and covariances on the dynamics of mean (global) quantities [5,11,16–18]; here again, the utility of this approach when the details at small scales are not known remains to be examined, as does the development of statistical methods to fit the models when data are only available for aggregated “mean” quantities.

## From Physiology to Ecosystem Dynamics: Global Change Ecology

The problem of incorporating sub-grid-scale processes into large-scale models is found in many other scientific fields in which nonlinearity allows variability to interact across spatial or organizational scales. It also applies to other ecological contexts, in particular to global change ecology and to the spatiotemporal ecosystem models used to represent feedbacks between the biota and the physical environment. At large spatial and temporal scales, the question of essential biological detail quickly becomes computationally intractable. In a recent review on ecosystem–atmosphere interactions, Moorcroft [3] emphasized the problem of scaling from the level of plant physiology to ecosystem-level dynamics to address global climate change questions. One limitation of existing approaches is that within the typical grid cell of existing models, different types of plants compete for resources that are highly homogeneous or averaged over space, generating a tendency for monocultures in the simulated biology [3]. This means that the fine-scale heterogeneity present in real plant communities, which is important for buffering the systems against perturbations, is lost [3,19,20]. Such heterogeneity is generated by small-scale ecological interactions among individuals and their interplay with stochastic physical disturbances, such as fire and gap formation from fallen adult trees. While models that simulate patterns of species community composition from individual plant interactions have been created, their application to ecosystem–atmosphere interactions is computationally prohibitive. To circumvent this problem, approximations to these individual-based models have been formulated in the form of size- and age-structured partial differential equations [21], which are close in spirit to the ideas discussed in the previous section (see also [22]).

**Figure 3:**
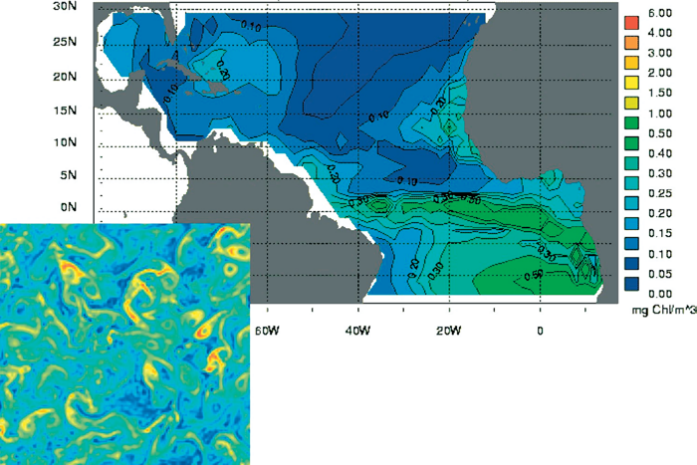
Phytoplankton Biomass Generated with a Coupled Biological–Physical Model Developed to Examine the Impact of Nitrogen Fixation in the Atlantic Ocean In this large simulation [24], the ecosystem model consists of six variables and includes two different functional groups within the phytoplankton, for nitrogen and non-nitrogen fixers. The physical model includes 19 vertical layers but only a coarse horizontal resolution (2° × 2°). In particular, it does not resolve the mesoscale variability of the flows, at characteristic scales of 1 to 100 km, known as the “weather” of the ocean. The lower left panel illustrates the variability of phytoplankton at these smaller turbulent scales, with a simulation of a coupled ecosystem–eddy model (K. Boushaba, G. Flierl, and M. Pascual, unpublished data). We can ask how the effects of these smaller scales can be incorporated in models with a coarser resolution for larger oceanic regions. Even more fundamentally, what are the relevant spatial scales of coupling?

Similar connections are being addressed in aquatic environments for phytoplankton, the unicellular primary producers of lakes and oceans estimated to contribute 45% of global net primary production (i.e., the amount of carbon fixed by plants per unit area over time via photosynthesis; E. A. Litchman, C. A. Klausmeier, J. R. Miller, O. Schofield, P. G. Falkowski, unpublished data). The amount of net primary production depends on biological heterogeneity in the form of a taxonomically diverse group of species [23]. A recently developed ecosystem model incorporates different phytoplankton functional groups and their competition for light and multiple nutrients (E. A. Litchman, C. A. Klausmeier, J. R. Miller, O. Schofield, P. G. Falkowski, unpublished data). Simulations of the model at specific sites to explore future scenarios suggest that global environmental change, including global-warming-induced changes, will alter phytoplankton community structure and hence alter global biogeochemical cycles (A. Litchman, C. A. Klausmeier, J. R. Miller, O. Schofield, P. G. Falkowski, unpublished data). The coupling of this type of ecosystem model to global climate models raises again a series of open questions on model complexity and relevant spatial scales of resolution. In fact, similar questions arise not just in the context of climate change but for the general coupling of ecosystem models to large-scale physical models of ocean circulation (e.g., [24]; Figure 3).

In short, the computational and conceptual challenge is to bridge not only highly disparate temporal and spatial scales, but also organizational ones, from individual physiology to ecosystem biogeochemistry, via community structure and functional diversity (Figure 3). An understanding of how the structure of ecological communities, composed of a diverse array of species, responds to perturbations is a critical intermediate step, which brings me to the next section.

## From Structure to Dynamics in Large Ecological Networks

The nonlinear dynamics of large networks is a major challenge in computational biology and complex systems in general [25]. In ecology, the study of networks of species interactions, particularly food webs composed of trophic links, has a long history at the interface of theory and empirical patterns [26–30]. Food webs are the subject of renewed attention today, with improved datasets and the explosion of research on network structure across science (see [31] for a review in ecology). However, the relationship between structure and dynamics in systems that are not just nonlinear and high-dimensional, but also adaptive, remains poorly understood. Food web structure includes the diversity of species, the patterns of connections among them, and the distribution of interaction strengths on these patterns; dynamics encompasses different measures of stability that describe the response of the system to perturbations such as robustness and resilience, which impact the persistence of species [32,33]. This is not a new area but many open questions remain [34]. For example, do rare species and those that interact weakly with others matter to overall species persistence in ecosystems? Simple models with only a few players suggest that rare or weakly interacting species can be important [35]; however, whether these findings still hold true in the bigger, more complex networks we observe in nature is not yet clear. Recent findings suggest a more complex picture in which not just the intensity of interactions, but also their location in the network, matters to (linear) stability and to the invasibility of the community by other species [36,37]. Another recent development is the consideration of other kinds of interactions such as mutualism and parasitism, which can play an important role in ecosystem persistence and bioenergetics [38,39]. The adaptive character of interactions via phenotypic plasticity and evolutionary change challenges traditional dynamical models and our view of structure itself. Adaptive change in the interactions between species influences dynamics and species' persistence, but again, this has been shown primarily in small networks with only a few players [40,41] (but see [42,43]). The spatial dimension has been largely ignored in the dynamics of large ecological networks, although it is clearly a key component of habitat loss and habitat fragmentation (but see [44] for a static treatment).

Stochastic assembly models are perhaps the best candidates to develop a general dynamical theory not only to address open questions on the relationship between structure and dynamics, but also to generate the macroscopic community patterns that ecologists observe in nature and characterize diversity (such as species–area curves and species-rank abundance curves). In these models, macroscopic patterns in diversity arise from the dynamic tension between extinction (as the result of ecological interactions and environmental perturbations) and innovation (as the result of evolution and the immigration of new species from outside the system) (e.g., [45]). Instability at one level of organization can provide the basis for robustness to change at higher levels. For example, in the species assembly model of Solé et al. [45], macroscopic quantities, such as the number of species, reach a stationary state, while at the microscopic level instability is rampant, with recurrent species extinctions and unpredictable population fluctuations. Computational developments are needed to tackle the large parameter space of this type of model and to study the model's behavior using methods that interface mathematical analysis and numerical simulation. Similar issues arise in complementary approaches to link structure and dynamics in food webs, including those that map nonlinear dynamical equations upon a static structure of links between species [46,47]. One promising direction to help us constrain parameter space and build more realistic models involves another fundamental area of ecology, the study of allometric scalings (e.g., [48]). Allometric scalings describe how biological rates vary as a function of size and can be used in the formulation of dynamical models for ecological interactions [49,50]. Ultimately, a better understanding of the critical properties of ecological networks that sustain diverse ecosystems and their functions is of fundamental importance, particularly at this time of rapid environmental change, when perturbations of structure and loss of biological diversity are unavoidable.

The topics described here only begin to illustrate some of the many rich areas for research in computational ecology. We have moved beyond learning more details about the components of complex systems in order to reconstruct their dynamics. Instead, a more fundamental role for computation is found in exploring the relationship between dynamics across scales, in the constant dialogue between simplicity and complexity. Perhaps this is best expressed by what a famous mathematician had to say about a famous macroscopic law of physics: “If our means of investigation became more and more incisive, we would discover the simple under the complex, then the complex under the simple, then again the simple under the complex, and so on, without being able to predict which state would ultimately prevail” [51]. 

## References

[pcbi-0010018-b01] Elton CS (1958). The ecology of invasions by animal and plants.

[pcbi-0010018-b02] Martinez ND (1991). Artifacts or attributes? Effects of resolution on the Little Rock Lake food web. Ecol Monogr.

[pcbi-0010018-b03] Moorcroft PR (2003). Recent advances in ecosystem–atmosphere interactions: An ecological perspective. Proc R Soc Lond B Biol Sci.

[pcbi-0010018-b04] Eames KT, Keeling MJ (2003). Contact tracing and disease control. Proc R Soc Lond B Biol Sci.

[pcbi-0010018-b05] Eames KT, Keeling MJ (2002). Modeling dynamic and network heterogeneities in the spread of sexually transmitted diseases. Proc Natl Acad Sci U S A.

[pcbi-0010018-b06] Meyers LA, Pourbohloul B, Newman MEJ, Skowronski DM, Brunham RC (2005). Network theory and SARS: Predicting outbreak diversity. J Theor Biol.

[pcbi-0010018-b07] Levin SA (1998). Ecosystems and the biosphere as complex adaptive systems. Ecosystems.

[pcbi-0010018-b08] Levin SA (1999). Fragile dominion: Complexity and the commons.

[pcbi-0010018-b09] Borges JL (1964). Dreamtigers. Boyer M, Morland H, translators.

[pcbi-0010018-b10] Durrett R, Levin SA (1994). On the importance of being discrete and (spatial). Theor Popul Biol.

[pcbi-0010018-b11] Keeling MJ (1999). Correlation equations for endemic diseases. Proc R Soc Lond B Biol Sci.

[pcbi-0010018-b12] Iwasa Y, Dieckmann U, Law R, Metz JAJ (2000). Lattice models in ecology and pair-approximation analysis. The geometry of ecological interactions: Simplifying ecological complexity.

[pcbi-0010018-b13] Pascual M, Mazzega P, Levin S (2001). Oscillatory dynamics and spatial scale in ecological systems: The role of noise and unresolved pattern. Ecology.

[pcbi-0010018-b14] Pascual M, Roy M, Franc A (2002). Simple models for ecological systems with complex spatial patterns. Ecol Lett.

[pcbi-0010018-b15] Gubbins S, Gilligan CA (1997). A test of heterogeneous mixing as a mechanism for ecological persistence in a disturbed environment. Proc R Soc Lond B Biol Sci.

[pcbi-0010018-b16] Bolker B, Pacala SW (1997). Using moment equations to understand stochastically driven spatial pattern formation in ecological systems. Theor Popul Biol.

[pcbi-0010018-b17] Pacala SW, Levin SA, Tilman D, Kareiva P (1997). Biologically generated spatial pattern and the coexistence of competing species. Spatial ecology: The role of space in population dynamics and interspecific interactions.

[pcbi-0010018-b18] Law R, Dieckmann U, Dieckmann U, Law R, Metz JAJ (2000). Moment approximations of individual-based models. The geometry of ecological interactions: Simplifying ecological complexity.

[pcbi-0010018-b19] Tilman D, Knops J, Wedin D, Reich P, Ritchie M (1997). The influence of functional diversity and composition on ecosystem processes. Science.

[pcbi-0010018-b20] Kinzig AP, Pacala SW, Tilman D (2001). The functional consequences of biodiversity: Empirical progress and theoretical extensions.

[pcbi-0010018-b21] Moorcroft PR, Hurtt GC, Pacala SW (2001). A method for scaling vegetation dynamics: The ecosystem demography model (ED). Ecol Monogr.

[pcbi-0010018-b22] Pascual M, Levin SA (1999). Spatial scaling in a benthic population model with density-dependent disturbance. Theor Popul Biol.

[pcbi-0010018-b23] Falkowski PG, Laws EA, Barber RT, Murray JW (2003). Phytoplankton and their role in primary, new, and export production In: Fasham MJR, editor.

[pcbi-0010018-b24] Coles VJ, Hood RR, Pascual M, Capone DG (2004). Modeling the impact of *Trichodesmium* and nitrogen fixation in the Atlantic Ocean. J Geophys Res.

[pcbi-0010018-b25] Strogatz SH (2001). Exploring complex networks. Nature.

[pcbi-0010018-b26] May RM (2001). Stability and complexity in model ecosystems, 1st Princeton landmarks in biology ed.

[pcbi-0010018-b27] Cohen JE (1978). Food webs and niche space.

[pcbi-0010018-b28] Yodzis P (1981). The stability of real ecosystems. Nature.

[pcbi-0010018-b29] Pimm SL (2002). Food webs.

[pcbi-0010018-b30] Polis GA, Winemiller KO (1996). Food webs: Integration of patterns and dynamics.

[pcbi-0010018-b31] Dunne JA, Pascual M, Dunne JA (2005). The network structure of food webs. Ecological networks: Linking structure to dynamics in food webs.

[pcbi-0010018-b32] Pimm SL, Lawton JH, Cohen JE (1991). Food web patterns and their consequences. Nature.

[pcbi-0010018-b33] Holling CS, Gunderson LH, Gunderson LH, Holling CS (2002). Resilience and adaptive cycles. Panarchy: Understanding transformations in human and natural systems.

[pcbi-0010018-b34] Pascual M, Dunne J, Levin SA, Pascual M, Dunne JA (2005). Challenges for the future: Integrating ecological structure and dynamics. Ecological networks: Linking structure to dynamics in food webs.

[pcbi-0010018-b35] McCann K, Hastings A, Huxel G (1998). Weak trophic interactions and the balance of nature. Nature.

[pcbi-0010018-b36] Kokkoris GD, Jansen VAA, Loreau M, Troumbis AY (2002). Variability in interaction strength and implications for biodiversity. J Anim Ecol.

[pcbi-0010018-b37] Neutel AM, Heesterbeek JAP, de Ruiter PC (2002). Stability in real food webs: Weak links in long loops. Science.

[pcbi-0010018-b38] Lafferty KD, Hechinger RF, Shaw JC, Whitney KL, Kuris AM, Collinge S, Ray C (2005). Food webs and parasites in a salt marsh ecosystem. Disease ecology: Community structure and pathogen dynamics.

[pcbi-0010018-b39] Bascompte J, Jordano P, Melián CJ, Olesen JM (2003). The nested assembly of plant–animal mutualistic networks. Proc Natl Acad Sci U S A.

[pcbi-0010018-b40] Hartvigsen G, Levin SA (1997). Evolution and spatial structure interact to influence plant–herbivore population and community dynamics. Proc R Soc Lond B Biol Sci.

[pcbi-0010018-b41] Peacor S, Riolo RL, Pascual M, Pascual M, Dunne JA (2005). Phenotypic plasticity and species coexistence: Modeling food webs as complex adaptive systems. Ecological networks: Linking structure to dynamics in food webs.

[pcbi-0010018-b42] McKane AJ, Drossel B, Pascual M, Dunne JA (2005). Models of food web evolution. Ecological networks: Linking structure to dynamics in food webs.

[pcbi-0010018-b43] Kondoh M (2003). Foraging adaptation and the relationship between food-web complexity and stability. Science.

[pcbi-0010018-b44] Brose U, Ostling A, Harrison K, Martinez ND (2004). Unified spatial scaling of species and their trophic interactions. Nature.

[pcbi-0010018-b45] Solé RV, Alonso D, McKane A (2002). Self-organized instability in complex ecosystems. Philos Trans R Soc Lond B Biol Sci.

[pcbi-0010018-b46] Chen X, Cohen JE (2001). Global stability, local stability and permanence in model food webs. J Theor Biol.

[pcbi-0010018-b47] Williams RJ, Martinez ND (2004). Stabilization of chaotic and non-permanent food-web dynamics. Eur Phys J B.

[pcbi-0010018-b48] Enquist BJ, Brown JH, West GB (1998). Allometric scaling of plant energetics and population density. Nature.

[pcbi-0010018-b49] De Leo GA, Dobson AP (1996). Allometry and simple epidemic models for microparasites. Nature.

[pcbi-0010018-b50] Emmerson MC, Rafaelli D (2004). Predator–prey body size, interaction strength and the stability of a real food web. J Anim Ecol.

[pcbi-0010018-b51] Poincaré H (1902). Science et hypothèse.

